# Supplementary material for: Assessment Tasks and Virtual Exergames for Remote Monitoring of Parkinson’s Disease: An Integrated Approach Based on Azure Kinect
Source: Sensors (Basel). 2022 Oct 25;22(21):8173. doi: 10.3390/s22218173 (PMC9654712; doi:10.3390/s22218173)
Supplement: Supplementary file 1 [file sensors-22-08173-s001.zip › Supplementary Materials/Supplementary Materials .pdf]

Supplementary Materials for "Assessment tasks and virtual exergames for remote monitoring of Parkinson's disease: an integrated approach based on Azure Kinect."

1. Exergames configuration parameters

The three exergames described in the paper can be configured according to a series of parameters set by clinicians. This allows proper tuning of the game according to the health status and the rehabilitation goals of the subject. Table S.1 reports the complete list with meaning. Each parameter has three possible values that can be selected by the clinician (EASY, MEDIUM, HARD) that are automatically mapped on real values used in the game (the unit is reported in the table as well).

Table S1. List of configuration parameters for virtual exergames.

Exergame	Parameter	Meaning	Unit
LWL/FWL	ARM_{MOV}	Number of movements to complete the game level	#
	ARM_{MINANG}	Minimum arm angle for "good" movements	deg
	$ARM_{MAXTIME}$	Maximum time to complete the level	sec
BB	LEG_{MOV}	Number of movements to complete the game level	#
	$BALL_{START}$	Ball starting position (percentage above knee rest position)	%
	$LEG_{MAXTIME}$	Maximum time to complete the level	sec
	$LIGHTON_{TIME}$	Time between a ball hit and consecutive light-on	sec

2. $INDEX_{SIM}$

The functional parameter $INDEX_{SIM}$ of LWL and FWL was defined based on the collected data to evaluate the time lag between left and right ANG_{ARM} signals at arm raise above ARM_{MINANG} (LAG_{OVER}) and descent below ARM_{MINAG} (LAG_{UNDER}). A simultaneous cycle is one for which the left arm and right arm are raised above ARM_{MINAG} such that one of the two motion cycles starts before the end of the other. Conversely, wrong single-arm movements are the ones for which is not possible to identify a corresponding raise cycle in the other arm signal before its end and are counted ($SINGLE_{COUNT}$) to be used as a penalty factor. The overall metric is mathematically defined as in the following Equation (S.1):

$$\mathrm{INDEX_{SIM}} = \left(\frac{\overline{LAG}_{OVER} + \overline{LAG}_{UNDER}}{2}\right) * penalty \qquad \text{Eq. (S.1)}$$

in which barred quantities are the mean values and the penalty factor is computed as

$$penalty = \begin{cases} SINGLE_{COUNT}, & SINGLE_{COUNT} > 0 \\ 1, & SINGLE_{COUNT} = 0 \end{cases}$$

As described in the paper, its value gets close to 0 for good time alignment in the relevant motion instants (crossing of the ARM_{MINANG} threshold), otherwise, it gets larger and larger according to the desynchronization level. Single arm raises, if present, penalize the result through amplification of the value ($penalty$).

3. LA vs BB in PD subjects

From the subject-by-subject comparison of responses in performing traditional Leg Agility (LA) and its gamified double, the Bouncing Ball exergame (BB), we have identified two relevant cases that well summarize our findings on the topic.
The first example is Subject 5 (Video S.1). Subject 5 performs almost identically in the two tasks; hence the configuration of the game well suits his performance and the subject himself does not alter his behavior during the task due to the gamified setting of BB.
The second relevant case is Subject 16 (Video S.2). Subject 16 follows two completely different strategies in the execution of the two tasks: in LA, a reduced amplitude, but the fast movement is performed with the leg involved in the execution. In BB, instead, the subject raises his leg well above the height required by the game to hit the ball, so the gamified setting stimulates the subject to overact, thus eliciting a truer estimation of the maximum height reachable with the knee. This aspect would not have been shown by the subject in a normal LA execution but was solicited by the game.
